# Quercetin protects cadmium-induced renal injuries in mice by inhibiting cell pyroptosis

**DOI:** 10.3389/fvets.2023.1319857

**Published:** 2023-11-16

**Authors:** Juyu Wang, Jieyan Yang, Kai Liu, Weijie Qu, Kuan Wang, Yu Zhao, Yaxiong Zhou, Xiang Liu, Limei Zhang, Xiaolong Gu

**Affiliations:** Department of Clinical Veterinary Medicine, College of Veterinary Medicine, Yunnan Agricultural University, Kunming, China

**Keywords:** cadmium, kidney injuries, oxidative stress, pyroptosis, quercetin

## Abstract

The toxic heavy metal cadmium (Cd) has a significant impact on kidney health. Documents manifested that non-toxic flavonoid quercetin can reduce Cd-induced kidney damage by reducing oxidative stress and inhibiting apoptosis, while the effect of quercetin on Cd-induced renal cell pyroptosis has not been elucidated. In this study, we established a model of Cd poisoning treated with quercetin both *in vitro* and *in vivo*. Results revealed that quercetin effectively reversed the decrease in Cd-induced cell viability. Furthermore, Cd increased blood urea nitrogen while reducing GPX and SOD levels, caused histopathological injuries in kidney with a significantly elevated cell pyroptosis characterized by enhanced levels of proteins representing assembly (NLRP3) and activation (pro IL-1β, cleaved IL-1β, and IL-18) of NLRP3 inflammasome as well as pyroptosis executor (pro caspase-1, cleaved caspase-1). However, quercetin administration alleviated kidney injuries above by decreasing cell pyroptosis. Overall, it suggests that kidney cells are susceptible to pyroptotic cell death due to Cd exposure; while quercetin exhibits protective effects through cell pyroptosis inhibition.

## 1 Introduction

Cadmium (Cd) is a silver-white heavy metal that is widespread in the environment. Cd pollution has the characteristics of a long half-life and long-lasting toxicity (1). Cd entering the body is mainly transported to various organs over the body through the blood and gradually accumulates, causing damage to multiple organs such as blood, heart, liver, kidneys, and testes in humans and animals. It is widely reported in populations and diverse experimental models that the kidney is a key target of chronic Cd exposure. Numerous studies have demonstrated that the pathological biochemical mechanism of renal damage caused by Cd is carried out mainly through the induction of oxidative stress (2). Cd indirectly produces reactive oxygen species (ROS) after entering human cells, alters renal endogenous antioxidant status, and causes peroxidative damage to biofilm lipids and DNA, eventually leading to apoptosis, has certain damage to the kidney (3, 4). Therefore, reducing the harm of Cd to the human body, especially the target organ kidney should be paid more and more attention. Numerous documents revealed that Cd could induce oxidative stress (5), pyroptosis (6), and autophagy in the kidney (7). Both pyroptosis and autophagy are programmed cell death that are completely different in morphology and mechanism. There was a study that showed Cd led to the upregulation of NLRP3, pro-caspase-1, caspase-1, IL-1β, and IL-18 in chicken livers (8), resulting pyroptosis.

Pyroptosis is involved in many pathophysiological processes including autoimmune and inflammatory diseases, infectious diseases, deafness, and cancer (9). The predominant characteristics of pyroptosis are perforation of the cell membrane and Caspase-1 dependent. Its primary induced-mechanism is considered to be the NLR pyrin domain containing 3 (NLRP3) inflammasomes, which can be triggered by different stresses (10). Upon inflammasome activation, cells undergo certain biochemical, and morphological changes, including processing of IL-1 family cytokines such as IL-1β and IL-18, changes in membrane shape including blebbing and ballooning, loss of membrane integrity, phosphatidylserine (PS) exposure on the outer plasma membrane, nuclear condensation, fragmentation of DNA, and disruption of certain organelles such as mitochondria and lysosomes (11). The NLRP3 inflammasomes recruit and bind to apoptosis-associated speck-like protein (ASC), resulting in ASC focus, which recruits pro-caspase-1 and activates Caspase-1, Caspase-1 involves in cleavage and maturation of pro-interleukin-18/1β (pro-IL-18/1β) and cleavage of gastrin D (GSDMD). Literature reported that GSDMD can be cleaved into the C-terminal domain and N-terminal domain of GSDMD (GSDMD-CT and GSDMD-NT) (12). GSDMD-NT can form pores in the cell membrane, then leading to IL-1β/IL-18 and soluble cytoplasmic content releases, including intracellular nitric oxide (NO) and lactate dehydrogenase (LDH), generating cell swelling and osmotic lysis. Once Caspase-1 is activated by NLRP3, pyroptosis will unavoidably happen. Importantly, a study revealed that a selective inhibitor of caspase-1 suppressed Cd-induced cell death (13).

Quercetin (Que) is a flavonoid compound, found in a variety of plants, fruits, Chinese medicine, and vegetables. Que has a strong anti-tumor effect, which exerts anti-cancer functions through cell signal transduction pathways such as antioxidation, anti-proliferation, and promotion of apoptosis (14, 15). In recent years, the protective effects of quercetin on toxicity induced by exogenous harmful substances have attracted increased attention (16). Studies have demonstrated that quercetin can prevent oxidative damage induced by Cd by scavenging oxygen free radicals, reducing lipid peroxidation, improving intracellular antioxidant status, and inhibiting apoptosis (17). In this work, we investigated the role of pyroptosis in Cd-induced kidney damage *in vitro* and *in vivo*. Additionally, we addressed the protected effect of quercetin against Cd-induced kidney injuries.

## 2 Materials and methods

### 2.1 Reagents

Creatinine (CRE, C011-2-1), Blood Urea Nitrogen (BUN, C013-2-1), Reduced glutathione (GSH, A006-2-1), and Malondialdehyde (MDA, A003-1-2) assay kits were obtained from Jiancheng Bioengineering Institute (Nanjing, China). Anhydrous CdCl_2_ (purity: >99.95%) was purchased from Aladdin Industrial Corporation. Que (purity: >97%) was obtained from RHAWN (Shanghai, China). β-Actin,α-tubulin and antibodies against cleaved caspase-1, pro-caspase-1, NLRP3, IL-1β, pro-IL-1β were obtained from Wanleibio (Shenyang, China).

### 2.2 Cell culture and treatment

MES-13 was obtained from Laboratory preservation. The MES-13 were cultured in DMEM supplemented with 10% FBS and 1% (v/v) penicillin/streptomycin at 37°C in a humidified atmosphere with 95% air and 5% CO_2_.

### 2.3 Experimental animals and exposure protocol

Six-week-old male C57BL/6 mice were purchased from Kunming and housed under standard environmental conditions with a room temperature of 22°C, humidity of 40–70%, and a 12:12 h light-dark cycle. All animal experimental processes were approved by the Institute of Zoology and Medical Ethics Committee of Yunnan Agricultural University and were strictly designed for animal welfare (approval number HAUST 20015). Forty mice were randomly divided into four groups with 10 mice per group. The experiment was carried out for 4 weeks. Mice body weight was recorded each week, and they were treated as follows. The control group was given daily saline (10 ml/kg). The Cd-treated group was injected with 1 mg/kg CdCl_2_ intraperitoneally. The Cd + Que group received 1 mg/kg CdCl_2_ by intraperitoneal injection and 50 mg/kg Que intragastrically. Que treatment group was administered orally with Que (50 mg/kg). The doses of Cd and Que were chosen based on previous studies. After 4 weeks, the mice were sacrificed under anesthesia and the kidneys were collected and immediately frozen in liquid nitrogen and stored at −80°C until analysis.

### 2.4 Cell viability assay

According to the manufacturer's instructions, cell viability was determined using the Cell Counting Kit-8 (CCK-8; Dojindo Kumamoto, Japan). Briefly, MES-13 was cultured in 96-well plates to reach the desired confluence. The cells were then incubated with different concentrations of CdCl_2_ (0, 5, 10, 20, 40, and 80 μM). After 12 and 24 h of CdCl_2_ treatment, concentration of CdCl_2_ (5 or 15 μM) at 80% cell viability was selected and co-treated with quercetin (0, 2, 5, 10, 20 μM) for 12 and 24 h, respectively. A number of 10 μL CCK-8 was added to each well. The cells were then incubated for 2 h at 37°C. Following incubation, the absorbance was read at 450 nm using a microplate reader (Tecan, Mannedorf, Switzerland).

### 2.5 Western blotting

Cellular and renal total proteins were extracted using RIPA lysis buffer containing 1 mM PMSF (Beyotime, Shanghai, China) and the protein concentration was determined using a BCA protein assay (Sharp, Shanghai, China). Thirty micrograms of total proteins were resolved on 12% SDS-PAGE gel and transferred to polyvinylidene difluoride membranes. Non-specific binding sites were blocked with 5% non-fat milk in TBST (100 mM Tris–HCl pH 7.4, 0.9% NaCl, and 0.1% Tween 20) at room temperature for 4 h. The membranes were washed with TBST and incubated with specific primary antibodies against NLRP3 (1:1,000), caspase-1 (1:1,000), cleaved-caspase-1 (1:500), IL-1β (1:1,000), cleaved-IL-1β (1:1,000), α-tubulin (1:5,000), β-actin (1:3,500) overnight at 4°C. The membranes were then incubated with anti-rabbit or anti-mouse HRP-conjugated IgG antibodies (1:2,000) for 2 h at room temperature. The protein bands were visualized by ECL™ and Western Blotting Detection Reagents (Pierce Chemical, Dallas, TX, USA). The densitometry was evaluated using Image J software.

### 2.6 Kidney function indexes

The venous blood of mice was collected and subsequently centrifuged at a speed of 3,000 r/min for 10 min to obtain the serum. BUN levels in the mouse serum were measured using kits with spectrophotometer, following the manufacturer's instructions. A total of 0.4 g kidney tissues were cut for measurement of antioxidant indexes, ground on ice at a ratio of kidney tissue (g) to saline = 1:9, and then centrifuged at 3,000 rpm for 10 min to obtain the supernatant. The levels of SOD and GPX in mouse kidney tissues were assessed using diagnostic kits with a spectrophotometer, according to the manufacturer's instructions.

### 2.7 Histopathological studies

The fresh, morphological, and structurally intact kidney tissues were fixed in 10% formalin for 48 h. And then it was rinsed with running water overnight and dehydrated with different concentrations of ethanol, transparent in xylene, and embedded in paraffin. The paraffin blocks were cut with a microtome to a thickness of 5 μM. The kidney sections were stained with hematoxylin-eosin and mounted for microscope observation.

### 2.8 Immunohistochemical fluorescence

The slides were dewaxed with xylene and rehydrated with graded ethanol for immunohistochemical analysis. After incubation with 3% hydrogen peroxide to block endogenous peroxidase, the slides were incubated with 10% goat serum for 30 min to block non-specific proteins. And then, the slides were incubated with specific primary antibodies at 4 °C overnight, followed by horseradish peroxidase-conjugated secondary antibody incubation for 30 min. Slides were then incubated with a developing solution (peroxidase substrate containing diaminobenzidine) with hematoxylin counterstaining. Lastly, images were captured. Brown signals, showing immunoreactivity, were analyzed using Image Pro Plus 6.0 (Media Cybernetics, Bethesda, MD, USA).

### 2.9 Statistical analysis

The results were was expressed as mean ± SEM. SPSS22.0 was used for statistical analysis. ANOVA was used to analyze the differences between multiple groups. LSD or Tamhane's T2 method was used following the homogeneity test of variance. The difference is statistically significant when *P* < 0.05 or *P* < 0.01.

## 3 Result

### 3.1 Cd cytotoxicity and protective effect of quercetin in MES-13

**Figure 1:**
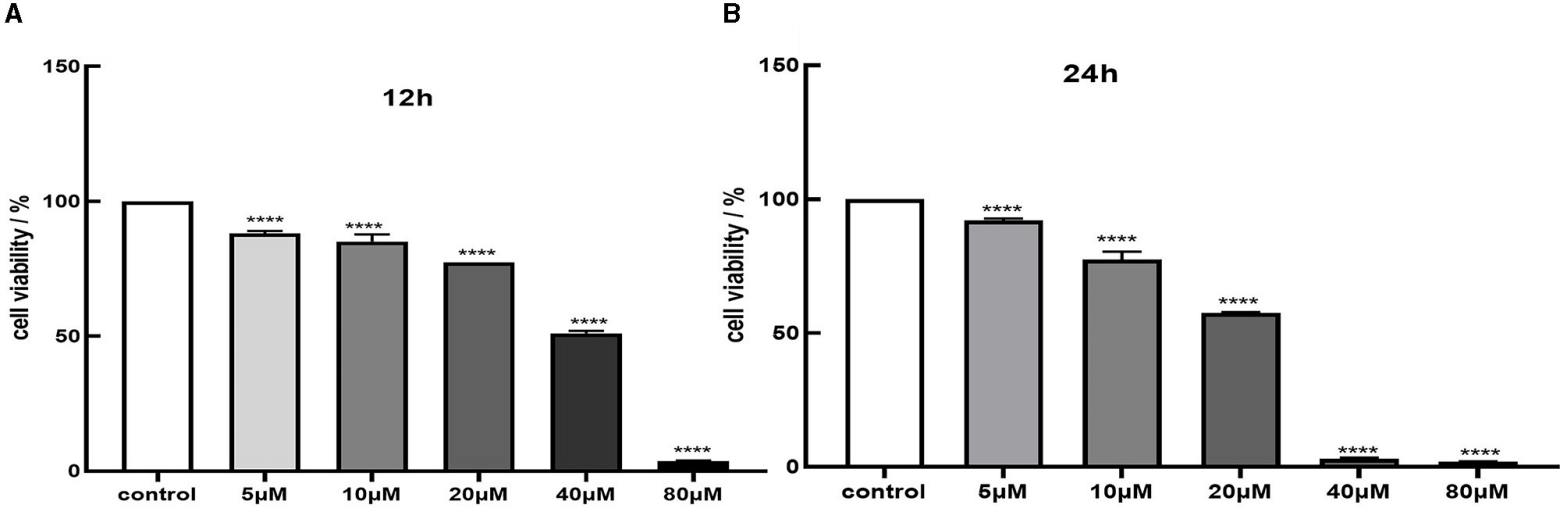
Cytotoxicity of Cd in MES-13 cells. MES-13 were exposed to different concentrations of CdCl_2_ for 12 h **(A)** and 24 h **(B)**. Cell viability was determined using the CCK-8 Test Kit. Values are mean ± SEM for three independent experiments. ****P < 0.0001 vs. the corresponding control group(con).

**Figure 2:**
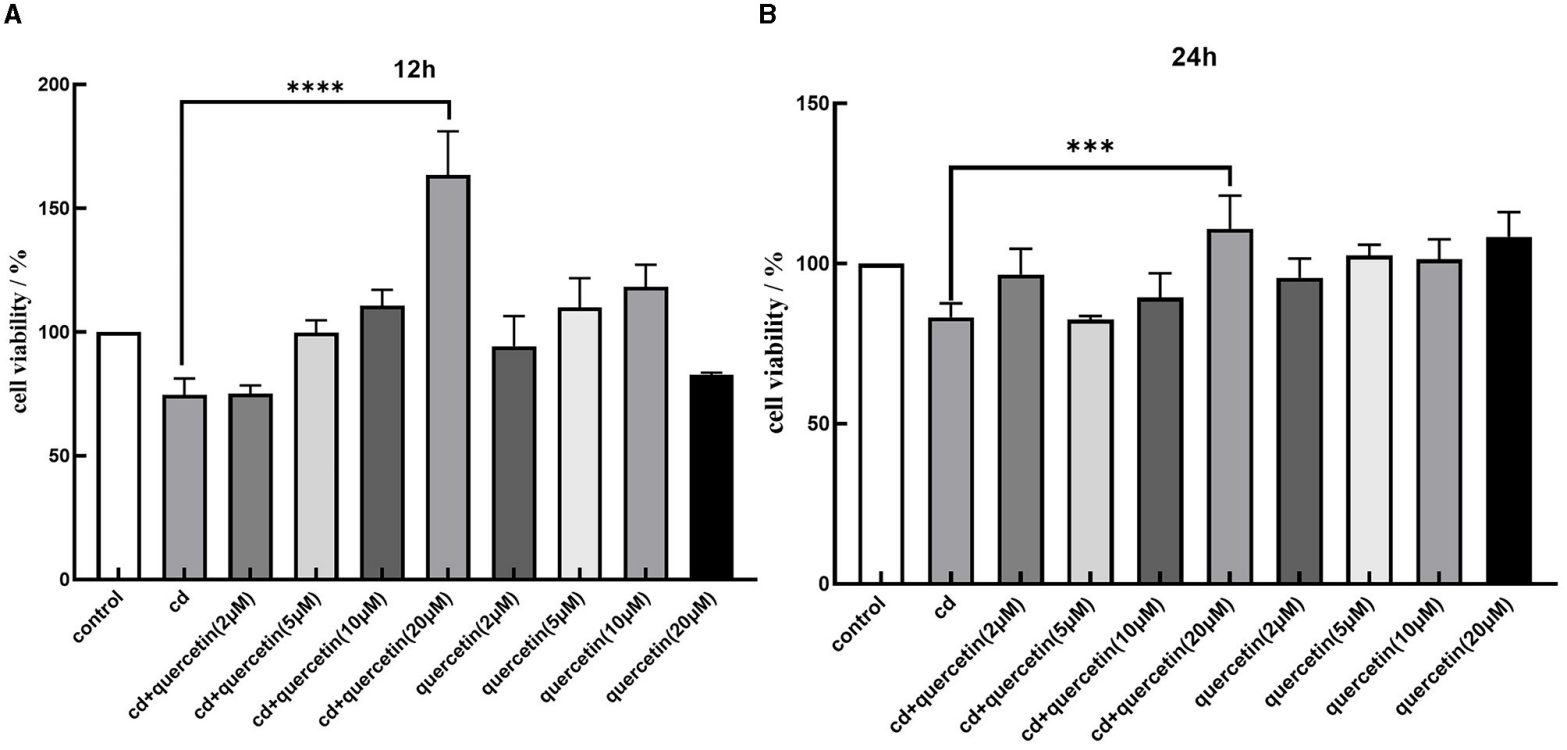
Effects of quercetin in Cd-induced cytotoxicity in MES-13 cells. MES-13 were exposed to different concentrations of quercetin and CdCl_2_ for 12 **(A)** and 24 h **(B)**. Cell viability was determined using the CCK-8 Test Kit. Values are mean ± SEM for three independent experiments. ***P < 0.001 and ****P < 0.0001 vs. the corresponding control group(con).

We assessed cell viability in MES-13 cells following exposure to various concentrations of CdCl_2_ (0, 5, 10, 20, 40, and 80 μM) for durations of 12 and 24 h. The results depicted in Figure 1 demonstrate a decline in the vitality of MES-13 cells with increasing concentrations of CdCl_2_ the 12 and 24 h incubation periods. These findings indicate that CdCl_2_ induces cytotoxicity in MES-13 cells in a dose-dependent and time-dependent manner. After treatment with CdCl_2_, cell viability decreased to ~80% compared to the control at concentrations of 15 μM for 12 h and 5 μM for 24 h. Consequently, we decided to utilize either 15 μM CdCl_2_ for a duration of 24 h 5 μM for a duration of 12 h as these conditions resulted in ~80% cell viability. Subsequently, different doses of quercetin (0, 2, 5, 10, and 20 μM) were individually combined with CdCl_2_ exposure. Figure 2 illustrates the administration of quercetin for a period of 2 h, followed by exposure to 15 μM CdCl_2_ for 12 h and 5 μM CdCl_2_ for 24 h. Quercetin effectively reversed the decrease in cell viability induced by both 5 μM CdCl_2_ and 15 μM CdCl_2_ treatments. After observing significant cytotoxicity induced by exposure to 5 μM CdCl_2_ for a period of 24 h, the co-treatment with 20 μM quercetin had remarkable therapeutic effects. This concentration was subsequently employed in future studies.

### 3.2 Quercetin inhibits Cd-induced change of pyroptosis-related indexes

**Figure 3:**
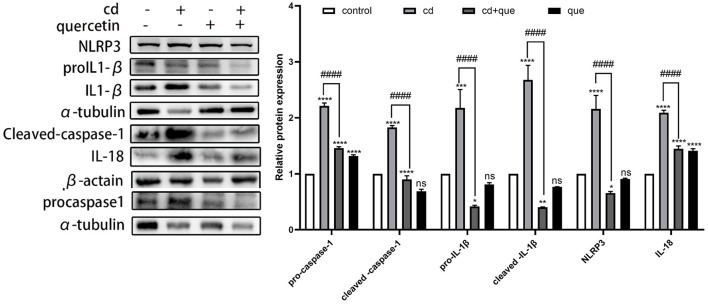
Effects of quercetin on the levels of pyroptosis-related proteins in Cd-induced cell pyroptosis in MES-13 cells. MES-13 cells were treated with 5 μM CdCl_2_ and 20 μM quercetin for 24 h. Western blotting was used to determine the amounts of pro- caspase-1, cleaved-caspase-1, pro-IL-1β, cleaved-IL-1β, IL-18, and NLRP3, with β-actin as a control. The results reflect the mean ± SEM of three separate experiments. *P < 0.05, **P < 0.01, ***P < 0.001, ****P < 0.0001 indicates an extremely significant difference compared to the control group; ^####^*P* < 0.0001 indicates a significant or extremely significant difference compared to the Cd group.

As shown in Figure 3, the levels of proteins representing assembly (NLRP3) and activation (pro IL-1β, cleaved IL-1β, and IL-18) of NLRP3 inflammasome as well as pyroptosis executor (pro caspase-1, cleaved caspase-1) in MES-13 cells were enhanced in CdCl_2_-exposed group (Figure 2B). Moreover, the assembly and activation of NLRP3 inflammasome as well as pyroptosis executor were reduced in Quercetin+Cd group, indicating that quercetin intervention was useful for the inhibition of kidney cell inflammation and pyroptosis. Briefly, these results suggest that CdCl_2_ induced NLRP3-dependent pyroptosis of kidney cells via upregulated IL-1β and IL-18 released from the pyroptotic cells promoted a series of pro-inflammatory responses and induce inflammation. Quercetin could alleviate pyroptotic cell death and pro-inflammatory response.

### 3.3 Cd causes kidney injuries and quercetin reduces kidney damage

**Figure 4:**
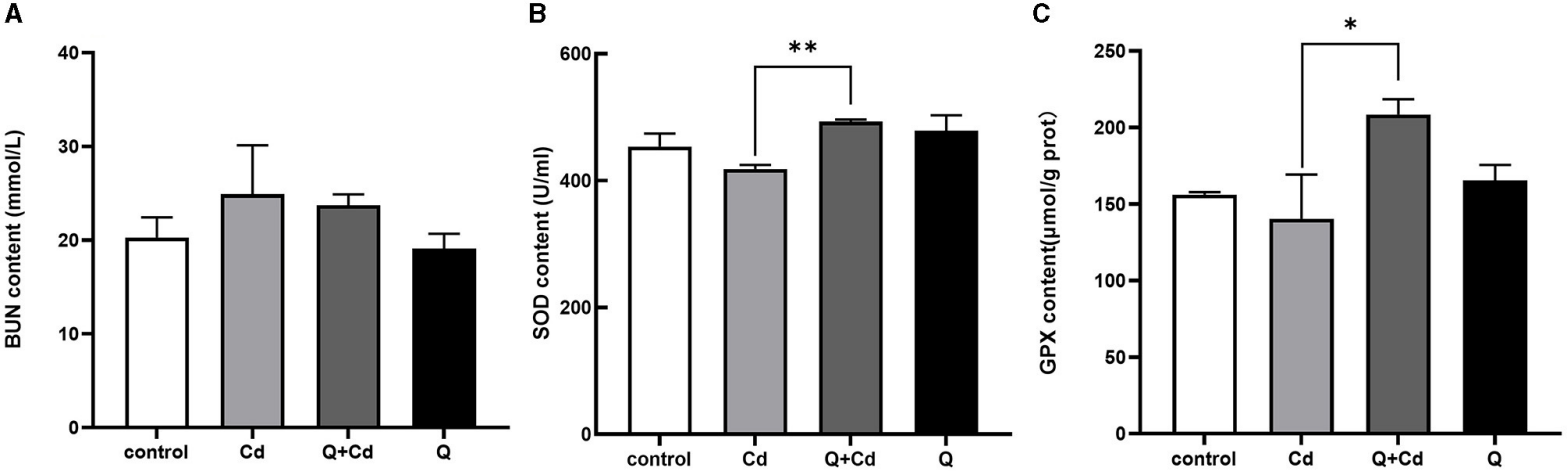
Effects of quercetin on renal function in Cd-induced kidney injuries in mouse. The content of BUN in serum **(A)**, the content of SOD in serum **(B)**, and the content of GPX in serum **(C)** were measured. The results reflect the mean ± SEM of three separate experiments. *P < 0.05, **P < 0.01 indicates a significant or extremely significant difference compared to the Cd group.

As shown in Figure 4, the levels of BUN in mice were enhanced in CdCl_2_-exposed group. The BUN levels with quercetin intervention were significantly reduced compared to those in the CdCl_2_ poisoning group. Additionally, in the Cd poisoning group, both the oxidative function indicators SOD and GPX were found to be reduced, whereas quercetin exhibited the ability to improve these indicators and enhance antioxidant capacity.

### 3.4 The effect of quercetin and cd on the histopathologic morphology of the kidney

**Figure 5:**
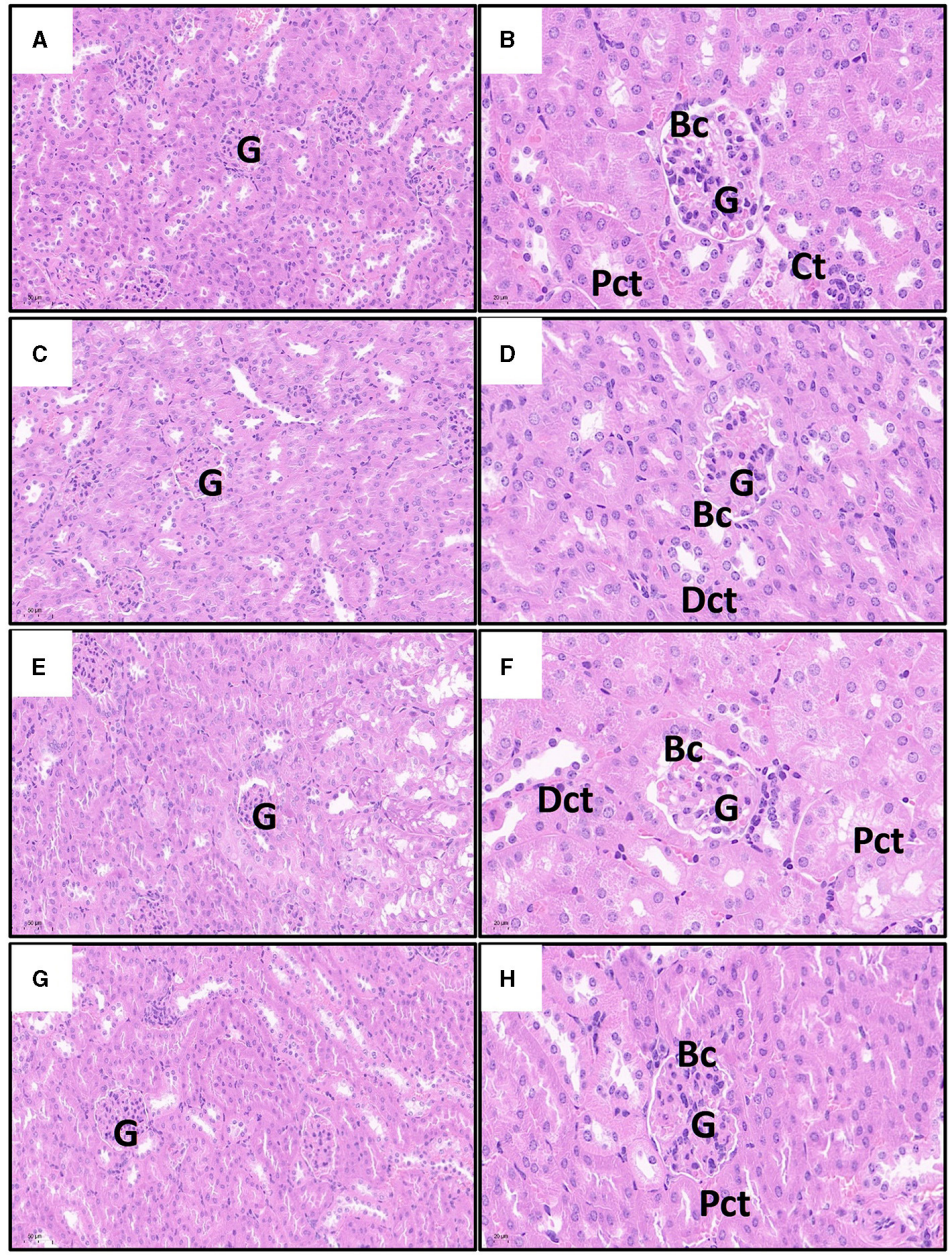
Effects of quercetin on changes in Cd-induced kidney histopathological injuries in mouse. The representative images of control group **(A)**, quercetin group **(C)**, Cd group **(E)**, and quercetin+Cd group **(G)** under lower magnification (scale bar = 50 μm). The representative images of control group **(B)**, quercetin group **(D)**, Cd group **(F)**, and quercetin+Cd group **(H)** under higher magnification (scale bar = 20 μm). G, glomeruli; Pct, proximal convoluted tubules; Dct, distal convoluted tubules degenerated; Bc, Bowman's capsule.

As shown in Figure 5, the kidney cells in the Cd poisoning group exhibited disorganized arrangement with indistinct cell borders in the pathological sections. Some cells underwent vacuolar degeneration, leading to disintegration and disappearance of their nuclei. The intercellular material became crowded, resulting in widened spaces between cells, while a few epithelial cells disintegrate and shed. Distinguishing individual renal tubules is challenging due to imperfect morphology and ambiguous boundaries between them. The structure of Bowman's capsule had transformed from a quasi-circular shape to an irregular one, and the demarcation line separating the glomerulus from the renal capsule is not clearly defined. In contrast, renal cells in control and quercetin groups with uniform cytoplasm distribution, prominent nuclei, consistent nuclear size, and well-defined cell boundaries. The boundaries between the renal tubules were clearly delineated, as is the lumen, and the structure of the glomerulus and Bowman's capsule appears normal. Furthermore, there is no evidence of interstitial congestion in both control and quercetin groups based on pathological sections. Quercetin treatment significantly mitigated renal tissue damage, interstitial histiocyte congestion, cell swelling, distinct cell boundaries, and clear gaps were presented between renal tubules. Moreover, the structure of the glomerulus and Bowman's capsule tended to exhibit normalcy.

### 3.5 The effect of quercetin on Cd-induced renal injuries

**Figure 6:**
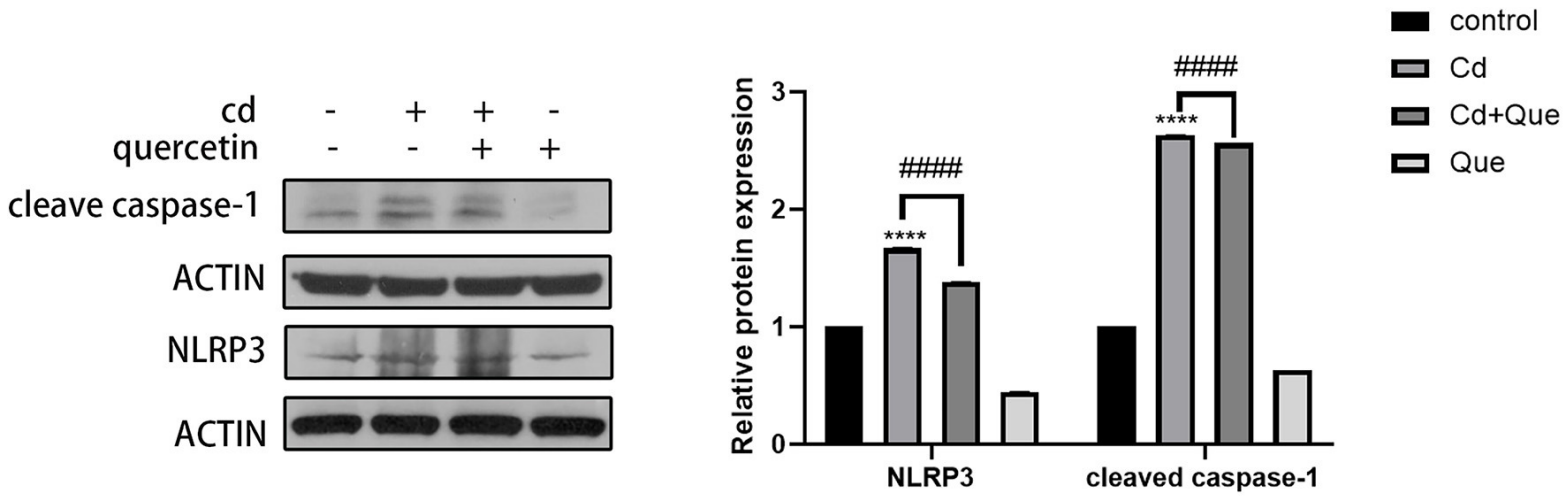
Effect of quercetin on cleaved-caspase-1 and NLRP3 in kidney tissues in Cd-induced kidney injuries in mouse. Western blotting was used to determine the amounts of cleaved-caspase-1 with β-actin as a control. The results reflect the mean ± SEM of three separate experiments. ****P < 0.0001 indicates an extremely significant difference compared to the control group; ^####^*P* < 0.0001 indicates a significant or extremely significant difference compared to the Cd group.

As shown in Figure 6, compared with the control group, the protein expression of cleaved caspase-1 and NLRP3 in the Cd-exposure group was significantly increased. Compared with the Cd poisoning group, quercetin intervention significantly reduced the protein expression of cleaved caspase-1 and NLRP3.

### 3.6 The effect of quercetin on Cd-induced renal cell pyroptosis

**Figure 7:**
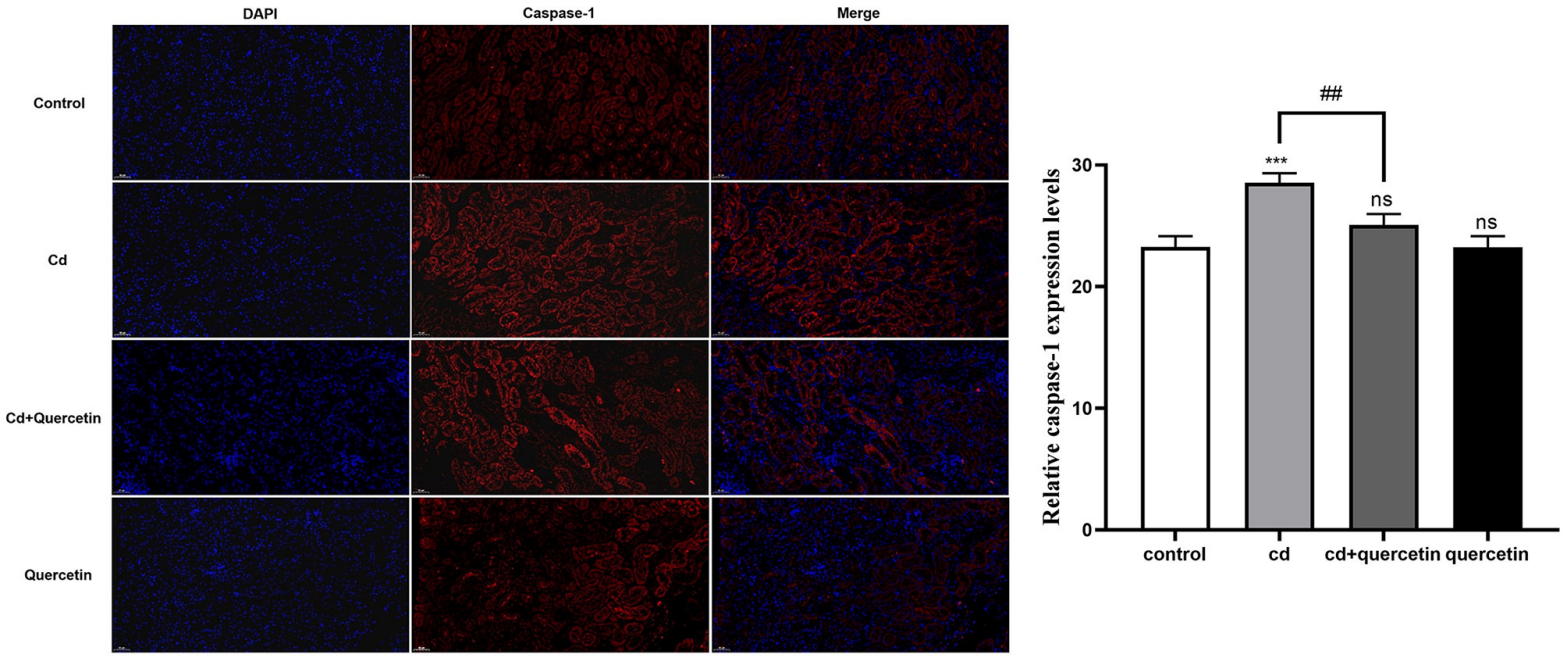
Effect of quercetin on cleaved-caspase-1 in kidney tissues in Cd-induced kidney injuries in mouse using Immunohistochemistry. Immunohistochemistry analysis was performed to detect the expression of cleaved caspase-1. The results were quantified and statistically analyzed. The results reflect the mean ± SEM of three separate experiments. ***P < 0.001 compared to the control group, ^##^*P* < 0.01 compared to the Cd group.

**Figure 8:**
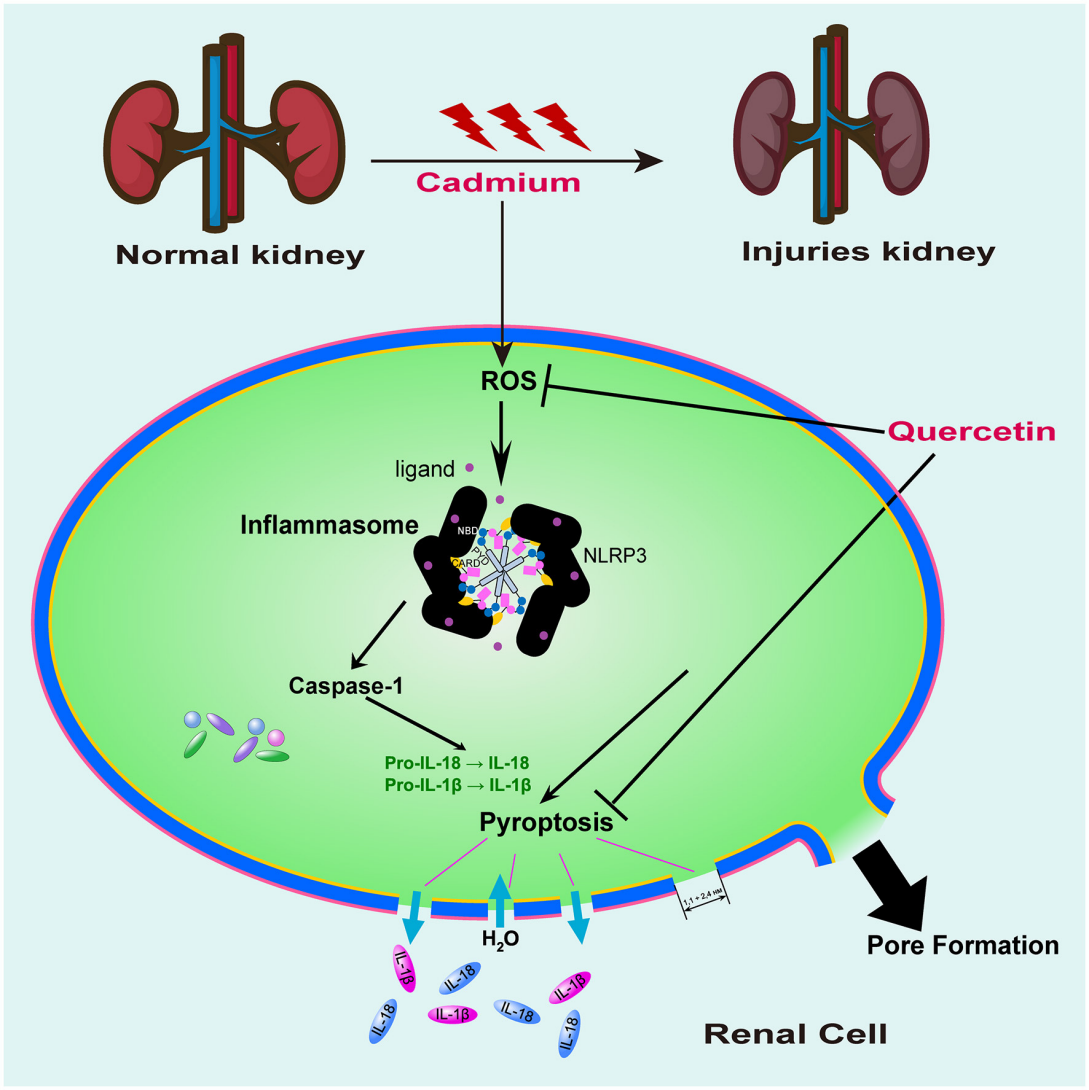
Proposed signaling pathway of quercetin on cadmium-induced kidney cell pyroptosis. Mouse kidney cell pyroptosis may be triggered by Cd exposure due to its promotion of ROS production resulting in oxidative damage to cells which activates caspase-1 for pyroptosis initiation. Quercetin exhibited antioxidant activity, effectively clearing ROS and inhibiting caspase-1 expression, thereby providing a protective effect against cell pyroptosis induced by cadmium exposure.

As depicted in Figure 7, kidney tissue slices from mice that had received CdCl_2_ injections were subjected to immunofluorescence analysis to further confirm the Cd-induced pyroptosis. While the amount of caspase-1 signals in the Cd -poisoned mice treated with quercetin was lower than that in the Cd-poisoned mice, the mice given CdCl_2_ showed an increase in the level of lysed caspase signal (red) in the marginal zone of the kidney compared with the control group. Schematic summary of the overall findings was shown in Figure 8.

## 4 Discussion

Cd is the most important heavy metal pollutant and kidney is the vital target organs of Cd toxicity (18). The proximal tubule of the kidney is the main site for Cd accumulation (19). Once the kidney is damaged, the glomerular filtration rate will decrease, resulting in increased BUN levels (20). The amount they excrete is closely related to glomerular filtration and renal tubular reabsorption (21). Luo et al. (22) found that Cd exposure raised the serum CRE and BUN at 1 mg/kg, suggesting kidney filtration failure. The study by Iserhienrhien et al. (23) also confirmed the damage of Cd exposure on rat renal function. In our study, BUN levels were elevated while SOD and GPX were found to be reduced in the Cd poisoning group, whereas quercetin exhibited the ability to reverse these changes.

Tang et al. (24) reported the induction of GSDME-mediated pyroptosis in triple-negative breast cancer cells (MDA-MB-231) upon acute Cd exposure, via ROS generation and NLRP3 inflammasome pathway activation. Another research manifested that Cd induces pyroptosis in a NLRP3 inflammasome-dependent manner and production of ROS in human dermal lymphatic endothelial cells (25). Both of the above studies reported the increased production of ROS upon Cd exposure; therefore, ROS generation might be a general mechanism following Cd exposure, as also reported in other cell types.

Numerous studies have linked Cd exposure to various health conditions including fatty liver, kidney disease, neurodegenerative diseases, and osteoporosis (26–28). However, the precise mechanism by which Cd induces these diseases remains unclear. Extensive research suggests that oxidative stress plays a crucial role in Cd-induced organ damage (29). The excessive production of reactive oxygen species (ROS) leads to oxidative damage, triggering apoptosis, pyroptosis, ferroptosis, and autophagy. Pyroptosis mainly depends on the formation of membrane pores by gastrin protein family members. The upstream mechanisms of activation of NLRP3 inflammasome include the production of ROS (30). Studies have shown that ROS produced by the NLRP3 activator acts as a second messenger whose signaling drives the activation of inflammasome (31). Caspase-1 is activated in or downstream of inflammasomes (32). Active caspase-1 induces pyroptosis, a necrotic form of regulated cell death characterized by plasma membrane permeabilization and rupture (33). In contrast to apoptosis, pyroptosis is considered to be a form of inflammatory cell death because pyroptotic cells release pro-inflammatory intracellular molecules, including interleukin-1 (IL-1) family cytokines and damage-associated molecular patterns (DAMPs), and caspases that induce pyroptosis (caspase-1/4/5/11) are called inflammatory caspases. Our findings revealed an increase in cleaved caspase-1 levels in mouse kidney tissue following Cd injection, furthermore, Cd exposure considerably elevated the levels of NLRP3 inflammasome assembly (NLRP3) and activation (pro IL-1β, cleaved IL-1β, and IL-18), as well as pyroptosis executor proteins (pro caspase-1, cleaved caspase-1) in renal cells. It is confirmed that Cd-induced pyroptosis is the key factor leading to kidney injury.

Quercetin, a typical flavonoid found abundantly in onions, peppers blueberries apples cherries, and other fruits and vegetables exhibits various physiological activities such as antioxidant properties anti-inflammatory immunomodulation abilities (34). In recent years, the protective effect of quercetin against toxicity induced by exogenous harmful substances has garnered increasing attention. Studies have demonstrated that quercetin can mitigate oxidative damage caused by Cd through scavenging oxygen free radicals, reducing lipid peroxidation, enhancing intracellular antioxidant status, and inhibiting cell apoptosis (16). However, most of these studies have primarily focused on the impact of quercetin in alleviating Cd-induced kidney injury and inhibiting apoptosis in rats, with few reports on the association between quercetin and necrotic cell death (35). In this study, we assessed indicators related to necrotic cell death and discovered that quercetin can exert a protective role by suppressing Cd-induced proptosis.

## 5 Conclusion

Results above demonstrated that mouse kidney cell pyroptosis may be triggered by Cd exposure due to its promotion of ROS production resulting in oxidative damage to cells which activates caspase-1 for pyroptosis initiation. Quercetin exhibits a certain level of protection against renal injury caused by Cd exposure. The findings suggested that quercetin might possess a protective influence on cadmium-induced nephrotoxicity by boosting the body's antioxidant defense mechanism and interfering with the pyrogenic signaling pathway.

## Data availability statement

The raw data supporting the conclusions of this article will be made available by the authors, without undue reservation.

## Ethics statement

The animal study was approved by Institute of Zoology and Medical Ethics Committee of Yunnan Agricultural University. The study was conducted in accordance with the local legislation and institutional requirements.

## Author contributions

JW: Data curation, Investigation, Methodology, Software, Writing—original draft. JY: Data curation, Investigation, Methodology, Software, Writing—original draft. KL: Data curation, Investigation, Methodology, Software, Writing—original draft. WQ: Data curation, Software, Writing—review & editing. KW: Funding acquisition, Resources, Writing—original draft. YuZ: Resources, Data curation, Writing—review & editing. YaZ: Data curation, Resources, Writing—review & editing. XL: Writing—review & editing, Project administration. LZ: Data curation, Investigation, Methodology, Software, Writing—original draft. XG: Data curation, Investigation, Methodology, Software, Writing—original draft, Writing—review & editing.
